# Patient‐Reported Symptoms and Mental Health Event Risks in Adolescents and Young Adults With Cancer

**DOI:** 10.1002/cam4.71096

**Published:** 2025-08-01

**Authors:** Sumit Gupta, Qing Li, Paul Nathan, Paul Kurdyak, Nancy Baxter, Rinku Sutradhar, Natalie Coburn

**Affiliations:** ^1^ Division of Haematology/Oncology The Hospital for Sick Children Toronto Canada; ^2^ Cancer Research Program ICES Toronto Toronto Canada; ^3^ Institute of Health Policy Management and Evaluation University of Toronto Toronto Canada; ^4^ Department of Paediatrics, Faculty of Medicine University of Toronto Toronto Canada; ^5^ Dalla Lana School of Public Health University of Toronto Toronto Canada; ^6^ Centre for Addiction and Mental Health CAMH Toronto Canada; ^7^ Li Ka Shing Knowledge Institute, St Michael's Hospital Toronto Canada; ^8^ Faculty of Medicine and Health University of Sydney Sydney Australia; ^9^ Department of Surgery Sunnybrook Health Sciences Centre Toronto Canada; ^10^ Department of Surgery University of Toronto Toronto Canada; ^11^ Sunnybrook Research Institute Toronto Canada

**Keywords:** adolescents and young adults, cancer, mental health, patient‐reported symptoms, population based, survivorship

## Abstract

**Introduction:**

Adolescents and young adults (AYA) with cancer are at risk of adverse mental health outcomes during and after treatment. Tools identifying AYA at the highest risk would guide screening and interventions. We determined whether self‐reported symptoms following cancer diagnosis were associated with early and late severe mental health events (SMHEs).

**Methods:**

Ontario AYA diagnosed with cancer aged 15–29 between 2010 and 2018 were identified and linked to healthcare databases, including one capturing self‐reported Edmonton Symptom Assessment System (ESAS) scores at cancer‐related visits. Scores for depression, anxiety, and poor well‐being were categorized as not measured, mild, moderate, or severe. SMHEs were defined as mental health‐related Emergency Department visits or hospitalizations. We determined the association of ESAS scores with subsequent early SMHEs (< 5 years). Among 5‐year survivors, we determined the association between the maximum ESAS score within 1 year of cancer diagnosis and late SMHEs (occurring > 5 years from cancer diagnosis).

**Results:**

Among 5435 AYA, symptom severity was associated with subsequent SMHE risk. AYA who reported severe versus mild anxiety were at > 3‐fold higher risk of subsequent early SMHEs [adjusted hazard ratio (aHR) 3.6, 95th confidence interval (CI) 1.9–6.7; *p* < 0.001]. Among 3518 (64.7%) 5‐year survivors, symptom severity predicted late SMHE. At 5 years postcancer diagnosis, those who reported severe versus mild depression within 1 year following cancer diagnosis were at 3‐fold elevated risk (aHR 3.0, 95 CI 1.8–4.9; *p* < 0.0001).

**Conclusion:**

Systematic symptom screening early postcancer diagnosis identifies AYA at high risk of both early and late SMHEs who may benefit from targeted screening and interventions.

## Graphical abstract

Adolescents and young adults (AYA) with cancer are at high risk of severe mental health events (SMHEs) during or after cancer treatment. Patient‐reported symptoms may identify those AYA at highest risk. Using population‐based data, we found that patient‐reported severe anxiety or depression within the first year of cancer diagnosis was strongly associated with both subsequent early SMHEs and late SMHEs occurring well into survivorship.

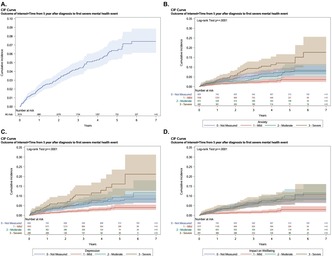

## 1 Introduction

Adolescents and young adults (AYA) with cancer are at risk of various adverse outcomes throughout the cancer journey [1]. Though adverse mental health outcomes both during and after cancer therapy are a challenge for all cancer patients and caregivers, AYA are at particularly high risk [2, 3, 4, 5, 6, 7]. For example, among a population‐based cohort of younger AYA with cancer in Ontario, 5‐year survivors experienced a 30% higher rate of mental‐health–related outpatient visits and a 20% higher risk of severe psychiatric episode as compared to age‐matched population controls [4]. How to best identify and help those AYA at the highest risk is unclear [8, 9].

Patient‐reported outcomes measures (PROMs) have gained prominence in the last decade, with multiple studies finding higher accuracy compared to healthcare provider assessments [10, 11]. The use of PROMs in clinical care to identify patients for various interventions can improve subsequent symptom burden and have sometimes been associated with better cancer outcomes [12, 13, 14]. A significant body of literature has examined patient‐reported distress among cancer patients using tools of varying complexity [9, 15, 16]. While a few studies have focused on the AYA cancer population, significant limitations remain. These studies have often involved small sample sizes, significant selection bias, and, when including clinical outcomes at all, the inability to collect longer term outcomes [9].

We aimed to overcome the above limitations by leveraging population‐based databases of both healthcare data and patient‐reported symptom scores to identify a cohort of AYA in Ontario, Canada. With this cohort, our objective was to determine whether patient‐reported symptom severity was associated with the subsequent risk of both early and late severe mental health outcomes.

## 2 Methods

### 2.1 Study Setting and Cohort

Canadian healthcare is delivered through provincial universal health insurance plans. Adult cancer care is available at both Regional Cancer Centers (RCCs) and community hospitals. Though individual institutions may have AYA‐focused programs, no provincial AYA cancer programs exist. In 2007, Ontario implemented provincial screening of patients at cancer‐related outpatient visits to optimize symptom control using the Edmonton Symptom Assessment System (ESAS), a validated patient‐reported measure assessing cancer‐associated symptoms including: pain, tiredness, drowsiness, nausea, lack of appetite, shortness of breath, depression, anxiety, and overall well‐being [17, 18]. Each is scored on an 11‐point scale from 0 (no symptoms) to 10 (worst possible symptoms) (Appendix 1), commonly categorized as no symptoms (0), mild (1–3), moderate (4–6), and severe (7–10) [19, 20]. Outpatient ESAS screening was broadly available at RCCs by 2010 [21, 22, 23]. Implementation among non‐RCCs was more variable. Scores are collected centrally in the Symptom Management Reporting Database (SMRD) [21, 22, 23, 24].

We used the Ontario Cancer Registry to create a retrospective population‐based cohort of all AYA diagnosed with a first cancer aged 15–29 years at diagnosis between January 1st, 2010 and June 30th, 2018 [24]. Populations who did not have access to ESAS were excluded: (1) those treated in pediatric institutions; (2) those not requiring RCC services in the first year postdiagnosis (e.g., thyroid cancer surgically resected in a community center with no additional therapy); and (3) those treated exclusively at non‐RCCs. Using unique encoded identifiers, patients were linked to population‐based health services databases (Table S1) held at ICES, a nonprofit research organization that is permitted by Ontario's privacy laws to use these data, without consent, for health system evaluation. The use of the data in this project is authorized under section 45 of Ontario's Personal Health Information Protection Act (PHIPA).

A subcohort was defined as those AYA who had survived at least 5 years from their original cancer diagnosis (i.e., survivor cohort).

### 2.2 Outcomes

Our main outcomes were early and late severe mental health events (SMHEs). SMHEs were defined as any mental health–related emergency department (ED) visit or hospitalization and were identified through validated algorithms using healthcare data (Appendix 2) [4, 25, 26, 27]. Early SMHEs were defined as occurring within the first 5 years postcancer diagnosis, while late SMHEs were defined as those occurring 5 years or later following the cancer diagnosis.

### 2.3 Predictor Variables and Covariates

Our key predictors of interest were self‐reported severity of three symptoms during the first year after cancer diagnosis: anxiety, depression, and poor well‐being. Symptom scores for each predictor were categorized as mild (0–3), moderate (4–6), severe (7–10), or not measured [24, 28, 29, 30]. Other predictors of interest related to mental health‐related healthcare use prior to cancer diagnosis. Through additional validated algorithms using billing codes, we identified any outpatient visit to a general practitioner for a mental health reason and all outpatient visits to psychiatrists that occurred between 27 and 3 months prior to the cancer diagnosis. We defined a binary covariate of whether or not a mental health–related outpatient visit had occurred during this 24‐month lookback window (yes vs. no) (Appendix 2) [4, 26, 27, 31, 32]. We also defined whether an SMHE had occurred during the 24‐month lookback window.

Covariates included demographic characteristics such as age at diagnosis (continuous) and sex. Neighborhood income quintile and urban/rural status were determined using Canadian census data [33, 34]. Regional location was categorized as the five main Ontario health regions (Central, East, North, Toronto, and West). Cancer type was categorized as hematologic, melanoma, central nervous system (CNS), sarcoma, testicular/ovarian, breast, colorectal, thyroid, and other. Time period of diagnosis was defined as early (2010–2014) and late (2015–2018).

### 2.4 Analyses

The primary analyses examined the association between ESAS score and (1) time to first early SMHE; and (2) late SMHE. For early SMHEs, the observation window started at the time of cancer diagnosis (index date). Patients were censored at the earliest of death, emigration from Ontario (two consecutive quarters of ineligibility for the Ontario Health Insurance Program), at 5 years postindex, or December 31st, 2020. The cumulative incidence function estimated the probability of each outcome over time. Cox regression estimated the association between symptom severity and each outcome, with ESAS score incorporated as a time‐varying four‐level categorical measure. If more than 28 days passed after an ESAS score without a subsequent measurement, patients were re‐categorized as “not measured.” Each ESAS symptom was considered separately. Unadjusted associations between ESAS score and time to early SMHE were determined and then adjusted for all covariates. Interaction terms between ESAS symptom severity and precancer diagnosis mental healthcare use were examined. Sensitivity analyses limited the observation window to either 1 or 3 years.

For late SMHEs, the observation window began at 5 years post the original cancer diagnosis (revised index date) with the maximum follow‐up to June 30, 2021. Only individuals who survived to this 5‐year mark were included in this analysis. The modeling strategy was the same as described above; however, ESAS symptom severity was conceptualized as a fixed four‐level categorical variable consisting of the maximum score noted during the first year following the cancer diagnosis.

To further explore the ability of self‐reported symptom burden and precancer diagnosis mental healthcare use to identify patients at the highest risk of late SMHEs, we determined the number of late SMHEs experienced by various subgroups of the cohort.

Statistical significance was defined as *p* < 0.05. Analyses were performed using SAS Enterprise Guide, version 7.15 (SAS Institute, Cary, NC). Ethics approval was obtained at The Hospital for Sick Children and Sunnybrook Health Sciences Centre. Informed consent was not required.

## 3 Results

After excluding those treated at pediatric centers, who did not require services at a cancer center in the first year after diagnosis, or who were exclusively seen at non‐RCCs, 5435 AYA remained of the original 9399 identified (Appendix 3) [24]. Cohort characteristics are shown in Table 1. The median follow‐up for the full cohort was 5.5 years [interquartile range (IQR) 3.3–8.1 years]. The median number of ESAS measurements per patient was 7 (IQR 2–15); the median number in the first year postcancer diagnosis was 3 (IQR 1–8).

**TABLE 1:** Distributions of demographic and disease characteristics of study cohort (*N* = 5435).

	*N* (%)
Age (years) (median, IQR)	25 (22–27)
Sex	
Male	2809 (51.7)
Female	2626 (48.3)
Time period	
Early (2010–2014)	3260 (60.0)
Late (2015–2018)	2175 (40.0)
Neighborhood income quintile	
Rural	535 (9.9)
Urban Q1 (lowest)	924 (17.0)
Urban Q2	1026 (18.9)
Urban Q3	952 (17.6)
Urban Q4	975 (18.0)
Urban Q5 (highest)	1009 (18.6)
Cancer type	
Hematologic	1748 (32.2)
Melanoma	343 (6.3)
CNS	335 (6.2)
Sarcoma	245 (4.5)
Testicular/Ovarian	1159 (21.3)
Breast	361 (6.6)
Colorectal	197 (3.6)
Thyroid	331 (6.1)
Other	716 (13.2)
Region	
Central	1737 (32.0)
East	1268 (23.3)
North	350 (6.4)
Toronto	585 (10.8)
West	1493 (27.5)

Abbreviations: CNS, central nervous system; IQR, interquartile range; N, number.

The cumulative incidence of SMHEs at 1 year was 2.8% (95 CI 2.4%–3.3%). Associations between symptom severity, previous mental healthcare use, and risk of early SMHE are shown in Table 2 (full model shown in Table S2), adjusted for sex, neighborhood income quintile/rurality, cancer type, region, time period of diagnosis, ESAS symptom severity, precancer diagnosis mental health‐related outpatient visit, and precancer diagnosis SMHE. In separate models, both moderate and severe anxiety or depression scores were strongly associated with subsequent SMHEs [severe vs. mild anxiety, adjusted hazard ratio (aHR) 3.6, 95 CI 1.9–6.7, *p* < 0.001; severe vs. mild depression, aHR 3.5, 95 CI 1.7–7.3, *p* < 0.001]. Previous mental healthcare use was also strongly associated; for example, AYA with a precancer diagnosis mental healthcare–related outpatient visit were nearly three times as likely to experience an early SMHE as those with no such visit (aHR across models 2.9, 95 CI 2.4–3.5; *p* < 0.001). Interaction terms between precancer diagnosis, mental healthcare use, and postcancer diagnosis symptom severity were not significant. Sensitivity analyses restricting the observation window to shorter time periods did not yield different results.

**TABLE 2:** Associations between time‐varying symptom severity, precancer diagnosis mental healthcare use, and early severe mental health event.

	Depression		Anxiety		Well‐being	
	Adjusted HR	*p*	Adjusted HR	*p*	Adjusted HR	*p*
Precancer diagnosis mental health–related outpatient visit						
Yes	2.9 (2.4–3.5)	< 0.001	2.9 (2.4–3.5)	< 0.001	2.9 (2.4–3.6)	< 0.001
No	Ref	—	Ref	—	Ref	—
Precancer diagnosis SMHE						
Yes	2.8 (2.2–3.8)	< 0.001	2.8 (2.2–3.8)	< 0.001	2.9 (2.2–3.8)	< 0.001
No	Ref	—	Ref	—	Ref	—
ESAS Score						
Not measured	1.3 (0.9–1.8)	0.17	1.3 (0.9–1.8)	0.17	1.2 (0.8–1.7)	0.32
Mild	Ref	—	Ref	—	Ref	—
Moderate	5.1 (3.1–8.5)	< 0.001	3.4 (2.0–5.8)	< 0.001	3.0 (1.9–4.9)	< 0.001
Severe	3.5 (1.7–7.3)	< 0.001	3.6 (1.9–6.7)	< 0.001	2.2 (1.0–4.9)	0.06

*Note:* Hazard ratios were determined through multivariable models including age, sex, neighborhood income quintile/rurality, cancer type, region, time period of diagnosis, ESAS symptom severity, previous mental health–related outpatient visit, and previous SMHE. Italicized hazard ratios indicate *p* < 0.05 while bolded hazard ratios indicate *p* < 0.001.

Abbreviations: HR, hazard ratio; SMHE, severe mental health event.

**FIGURE 1:**
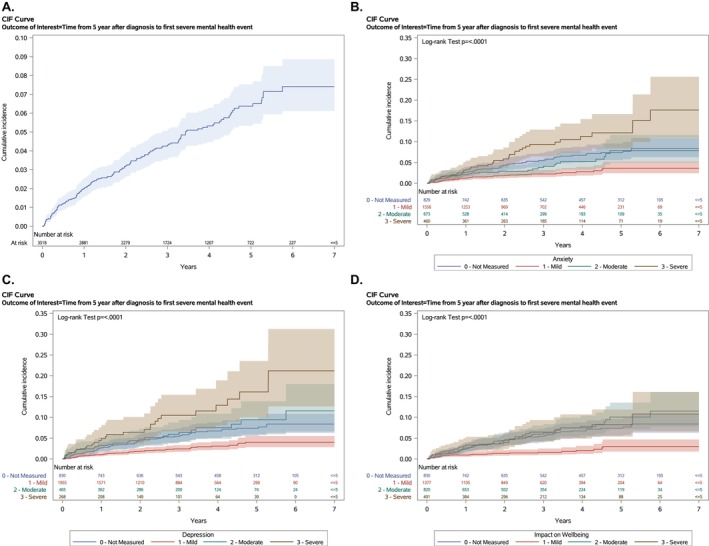
Cumulative incidence of late severe mental health events among 5‐year survivors.

A total of 3518 AYA had at least 5 years of follow‐up and were included in the late SMHE cohort. Characteristics are shown in Table S3. The median follow‐up for this subcohort was 2.9 years from the point of 5‐year survivorship (IQR 1.4–4.6 years). The cumulative incidence of late SMHEs (starting at 5 years from the cancer diagnosis) was 6.4% (95 CI 5.4%–7.6%) at 5 years (i.e., 10 years from the cancer diagnosis) (Figure 1A). Both maximum symptom burden in the first year following cancer diagnosis and precancer diagnosis mental healthcare use were highly associated with the risk of late SMHEs (Table 3, Table S4). For example, patients who reported severe anxiety at any point in the first year following cancer diagnosis had nearly three times the hazard for a late SMHE than those who had only reported mild anxiety (aHR 2.8, 95 CI 1.7–4.6, *p* < 0.001). Interaction terms between precancer mental healthcare use and postcancer symptom severity were not significant. The cumulative incidence of late SMHEs by maximum symptom severity is illustrated in Figure 1B–D.

**TABLE 3:** Associations between maximum symptom severity within 1‐year of cancer diagnosis, precancer diagnosis mental healthcare use, and late severe mental health event.

	Depression		Anxiety		Well‐being	
	Adjusted HR	*p*	Adjusted HR	*p*	Adjusted HR	*p*
Precancer diagnosis mental health–related outpatient visit						
Yes	1.9 (1.3–2.8)	< 0.001	2.0 (1.4–2.9)	< 0.001	2.1 (1.5–3.0)	< 0.001
No	Ref	—	Ref	—	Ref	—
Precancer diagnosis SMHE						
Yes	4.2 (2.6–6.7)	< 0.001	4.2 (2.6–6.7)	< 0.001	4.3 (2.7–6.8)	< 0.001
No	Ref	—	Ref	—	Ref	—
ESAS Score						
Not measured	1.8 (1.2–2.8)	0.004	1.9 (1.2–2.9)	0.006	2.4 (1.5–4.1)	< 0.001
Mild	Ref	—	Ref	—	Ref	—
Moderate	2.4 (1.5–3.9)	< 0.001	1.6 (1.0–2.7)	0.07	3.2 (1.9–5.2)	< 0.001
Severe	3.0 (1.8–4.9)	< 0.001	2.8 (1.7–4.6)	< 0.001	2.6 (1.5–4.5)	0.001

*Note:* Hazard ratios were determined through multivariable models including age, sex, neighborhood income quintile/rurality, cancer type, region, time period of diagnosis, ESAS symptom severity, previous mental health‐related outpatient visit, and previous SMHE. Italicized hazard ratios indicate *p* < 0.05 while bolded hazard ratios indicate *p* < 0.001.

Abbreviations: HR, hazard ratio; SMHE, severe mental health event.

The proportion of late SMHEs experienced by various subgroups of the cohort defined by symptom severity within a year of cancer diagnosis and by precancer diagnosis mental healthcare use is shown in Table 4 (full data in Table S5). For example, the 460 AYA who endorsed severe anxiety following their cancer diagnosis represented 13.1% of the cohort but accounted for 23.8% of AYA who experienced SMHEs during the first 3 years of survivorship. Patients who had both accessed an outpatient mental health visit prior to cancer diagnosis and endorsed severe anxiety (*N* = 157) represented only 4.5% of the cohort but accounted for 17.5% of AYA who experienced late SMHEs.

**TABLE 4:** Percentage of late SMHE accounted for by cohort subgroups.

Symptom burden	Precancer diagnosis outpatient mental health use	Precancer diagnosis SMHE	Percentage of full cohort	Percentage of all late SMHE
A. Anxiety				
Not severe	—	—	86.9	76.2
Severe	—	—	13.1	23.8
Not severe	No	—	70.7	45.8
Not severe	Yes	—	16.3	28.3
Severe	No	—	8.6	8.3
Severe	Yes	—	4.5	17.5
Not severe	—	No	84.3	59.2
Not severe	—	Yes	2.6	15.0
Severe	—	No	12.3	20.8
Severe	—	Yes	0.8	5.0

Symptom burden	Precancer diagnosis outpatient mental health use	Precancer diagnosis SMHE	Percentage of full cohort	Percentage of all late SMHE
B. Depression				
Not severe	—	—	92.4	82.5
Severe	—	—	6.7	17.5
Not severe	No	—	74.8	48.3
Not severe	Yes	—	17.6	34.2
Severe	No	—	4.5	5.8
Severe	Yes	—	3.1	11.7
Not severe	—	No	89.6	67.5
Not severe	—	Yes	2.8	15.0
Severe	—	No	7.0	12.5
Severe	—	Yes	0.6	5.0

*Note:* Full data shown in Table S5. ^a^In this table, defined as SMHE occurring between 5 and 8 years following cancer diagnosis. For example, patients reporting severe anxiety in the first year postcancer diagnosis and with a history of precancer diagnosis outpatient mental health use accounted for 4.5% of the full cohort but 17.5% of the late SMHE occurring between 5 and 8 years following cancer diagnosis.

Abbreviation: SMHE, Severe mental health event.

## 4 Discussion

In this population‐based cohort of over 5000 AYA with cancer, we found that self‐reported severity of anxiety and depression was strongly associated with subsequent early SMHEs, even when accounting for patient‐ and disease‐related factors such as precancer diagnosis mental healthcare use. Self‐reported severity of the same symptoms in the first year after cancer diagnosis also strongly predicted late SMHEs that occurred 5 years after the cancer diagnosis or later.

Previous work by our group and others has demonstrated that survivors of AYA cancer are at elevated risk for adverse mental health outcomes during and after cancer treatment [2, 3, 4, 5, 6, 7]. The risk of a major depressive disorder, as identified through claims data, was over three‐fold higher among over 3000 Japanese survivors of AYA cancer than among controls [35]. Using community health surveys, a Canadian group found that AYA cancer survivorship was associated with a doubling in the prevalence of both mood and anxiety disorders [5]. Identifying those AYA at highest risk would be of significant utility, allowing limited resources for screening and intervention to be targeted appropriately. However, identifying factors associated with poor mental health in this population has been difficult, with female sex, younger age, cancer type, and treatment modality showing inconsistent results [8].

Identifying symptoms through PROMs, versus healthcare provider assessment, may increase quality of life among cancer patients through adequate symptom control, and in some cases, decrease healthcare utilization and improve clinical outcomes [12, 13, 14, 36, 37]. Though a rich literature exists studying psychosocial and distress screening among cancer patients, some specific to the AYA population, reported validity and effectiveness have varied, with significant heterogeneity in the specific measures used, frequency of screening, link to interventions, and populations studied [9, 15, 16]. Some authors have suggested that shorter and/or simpler tools, such as the Distress Thermometer, may have similar validity as longer and more complex ones, while still noting methodological limitations to the existing literature [38, 39]. Our study makes a significant addition to this literature by examining the potential utility of simple PROMs, which combine measures of mental and physical symptoms to predict both early and late clinically meaningful mental health outcomes.

We demonstrate that, in the context of an organized provincial system for symptom screening, self‐reported severity of symptoms such as depression and anxiety is strongly associated with increased risk of subsequent early SMHEs (mental health–related ED visits or hospitalizations). In a previous Ontario study, ESAS score was also found to predict nonfatal self‐injury within 180 days among a general adult cancer population [30]. Whether ESAS‐based automatic triggers for interventions such as referral to social workers, psychologists, or psychiatrists would mitigate this risk is still unclear, but of significant potential impact on AYA patients and caregivers [16].

Perhaps surprisingly, self‐reported severity of the same symptoms was also strongly predictive of late SMHEs, despite long latency between the reported symptoms (first year following cancer diagnosis) and the severe events (5 years following cancer diagnosis or later). This finding provides evidence for the significant and long‐lasting psychological impact of an AYA cancer diagnosis, but also suggests that the simple ESAS tool can identify patients who may benefit from intensified mental health screening even after the end of cancer‐directed therapy. The lack of statistically significant interactions between symptom severity and precancer diagnosis mental healthcare use suggests that ESAS screening has utility in patients with and without a previous mental health history. For example, patients with such a history who also self‐reported severe anxiety comprised only 4.5% of the cohort but accounted for 17.5% of AYA who experienced SMHEs between 5 and 8 years following cancer diagnosis.

Whether psychosocial support during treatment is associated with improved long‐term mental health is unknown. Previous work has shown that survivors of AYA cancer who were originally treated at adult centers experienced 80% higher rates of mental health–related outpatient visits compared to similarly aged AYA who were originally treated at pediatric centers, despite the latter having already transitioned into the adult healthcare system [4]. While the authors speculated that this might be attributed to more intensive psychosocial care available in pediatric institutions, they were not able to study this hypothesis. While our findings show that it is possible to identify at‐risk AYA cancer survivors, such identification is only clinically significant should interventions exist that can prevent a meaningful proportion of these adverse events. Of note, any such intervention would not only be highly valued by patients, but has the potential to be cost‐effective given the impact of mental health on quality of life and productivity [40, 41]. Our results provide a population of AYA cancer survivors who should be targeted in trials of such interventions.

Designing such interventions required understanding the mechanisms underlying these associations and risks, which are likely multifactorial. Many AYA cancer survivors experience fear of recurrence even after the time of greatest risk has passed [42, 43, 44]. Financial toxicity may result in longer‐term socioeconomic challenges with attendant mental health impacts [45, 46]. Survivors of AYA cancer may also suffer from physical long‐term effects of their cancer or cancer therapy, leaving them with chronic morbidities and functional limitations, which in turn may negatively impact mental health [47, 48]. The relative importance of these and additional mechanisms may vary between patients, complicating the design of potential interventions.

Study strengths include the real‐world population‐based nature of our cohort, large sample size despite maintaining a focus on AYA, availability of prospective patient‐reported symptom scores, and ability to capture late mental health outcomes. However, several limitations also merit note. First, while we focused on ED visits and hospitalizations as a marker of severe mental health distress, we did not capture a broader range of adverse mental health that required only outpatient visits, or indeed patients suffering from adverse mental health who did not access the healthcare system. Second, our prior work has noted that certain subpopulations of AYA are less likely to participate in ESAS screening, such as those living in lower income urban neighborhoods, possibly limiting the generalizability of our findings to those groups [24]. Third, race and ethnicity variables were not available in our datasets. Fourth, detailed treatment‐related information, including duration of treatment, was not available. Our study, thus, cannot determine whether screening AYA during versus off treatment may have different ability to identify those at highest risk of early or late SMHEs.

In conclusion, systematic symptom screening in the first year after cancer diagnosis identifies a proportion of AYA at high risk of not only early SMHEs, but also continued late risk after the completion of cancer therapy. Future work is warranted to study how to best mitigate their risk through targeted screening and interventions both during and after cancer therapy.

## Author Contributions

**Sumit Gupta:** conceptualization (equal), funding acquisition (equal), methodology (equal), writing – original draft (equal), writing – review and editing (equal). **Qing Li:** formal analysis (equal), writing – review and editing (equal). **Paul Nathan:** conceptualization (equal), writing – review and editing (equal). **Paul Kurdyak:** supervision (equal), writing – review and editing (equal). **Nancy Baxter:** conceptualization (equal), funding acquisition (equal), supervision (equal), writing – review and editing (equal). **Rinku Sutradhar:** conceptualization (equal), formal analysis (equal), funding acquisition (equal), methodology (equal), supervision (equal), writing – review and editing (equal). **Natalie Coburn:** conceptualization (equal), methodology (equal), supervision (equal), writing – review and editing (equal).

## Conflicts of Interest

The authors declare no conflicts of interest.

## Supporting information


Data S1.

## Data Availability

Ontario privacy disclosure does not allow the sharing of personal health information; study data are thus not publicly available.
