# Supplementary material for: Patient‐Reported Symptoms and Mental Health Event Risks in Adolescents and Young Adults With Cancer
Source: Cancer Med. 2025 Aug 1;14(15):e71096. doi: 10.1002/cam4.71096 (PMC12314545; doi:10.1002/cam4.71096)
Supplement: Supplementary file 1 — Data S1. [file CAM4-14-e71096-s001.docx]

Supplemental Table 1. Population-based health services databases used in this study

| **Database** | **Data Elements** | **Description** | **Initiation Year** |
| --- | --- | --- | --- |
| ALR | Cancer system therapies | Patient-level activity within the cancer system, focused on radiation therapy and systemic therapies, including chemotherapy. Also captures outpatient oncology visits | 2005 |
| DAD | Inpatient hospitalizations | One record per hospital admission including chart-abstracted demographic, clinical and outcome data | 1988 |
| NACRS/SDS | ED visits/Same day surgery | Demographic, clinical and disposition data | 2000 |
| OCR | Cancer diagnoses | One record per new cancer diagnosis | 1964 |
| OHIP | Physician claims | Claims for services billed by fee-for-service Ontario physicians. Physicians under alternative funding plans are also required to submit shadow claims, ensuring capture of nearly all physician encounters | 1991 |
| OMHRS | Inpatient hospitalizations | One record per hospitalization to any inpatient mental health designated bed | 2005 |
| SMRD | Symptom scores | Patient-reported symptom burden | 2007 |

ALR – Activity Level Reporting; DAD – Discharge Abstract Database; ED – emergency department; NACRS – National Ambulatory Care Reporting System; OCR – Ontario Cancer Registry; OHIP – Ontario Health Insurance Plan Claims Database; OMHRS – Ontario Mental Health Reporting System; SMRD – Symptom Management Reporting Database; SDS – Same Day Surgery

Supplemental Table 2. Multivariable predictors of early severe mental health events

|  | Depression | | Anxiety | | Wellbeing | |
| --- | --- | --- | --- | --- | --- | --- |
|  | Adjusted HR | P-value | Adjusted HR | P-value | Adjusted HR | P-value |
| Age (per 10 years) | **0.6 (0.5-0.8)** | **<0.001** | **0.6 (0.5-0.8)** | **<0.001** | **0.6 (0.5-0.8)** | **<0.001** |
| Sex |  |  |  |  |  |  |
| Male | Ref | - | Ref | - | Ref | - |
| Female | 0.9 (0.8-1.1) | 0.48 | 0.9 (0.8-1.1) | 0.46 | 0.9 (0.8-1.1) | 0.50 |
| Time period |  |  |  |  |  |  |
| Early (2010-2014) | 0.9 (0.8-1.1) | 0.50 | 0.9 (0.8-1.1) | 0.51 | 0.9 (0.8-1.1) | 0.51 |
| Late (2015-2018) | Ref | - | Ref | - | Ref | - |
| Neighborhood income quintile |  |  |  |  |  |  |
| Rural | *1.5 (1.1-2.1)* | *0.02* | *1.5 (1.1-2.1)* | *0.02* | *1.5 (1.1-2.1)* | *0.02* |
| Urban Q1 (lowest) | 1.2 (0.9-1.7) | 0.18 | 1.2 (0.9-1.7) | 0.18 | 1.2 (0.9-1.7) | 0.17 |
| Urban Q2 | 1.2 (0.9-1.7) | 0.18 | 1.2 (0.9-1.7) | 0.18 | 1.2 (0.9-1.7) | 0.17 |
| Urban Q3 | 1.1 (0.8-1.5) | 0.51 | 1.1 (0.8-1.5) | 0.51 | 1.1 (0.8-1.5) | 0.51 |
| Urban Q4 | 1.1 (0.8-1.6) | 0.39 | 1.1 (0.8-1.6) | 0.39 | 1.1 (0.8-1.6) | 0.40 |
| Urban Q5 (highest) | Ref | - | Ref | - | Ref | - |
| Cancer type |  |  |  |  |  |  |
| Hematologic | 1.0 (0.7-1.5) | 0.94 | 1.0 (0.7-1.5) | 0.95 | 1.0 (0.7-1.5) | 0.95 |
| Melanoma | 1.2 (0.7-1.9) | 0.59 | 1.1 (0.7-1.9) | 0.61 | 1.2 (0.7-1.9) | 0.58 |
| CNS | 1.4 (0.9-2.3) | 0.18 | 1.4 (0.9-2.4) | 0.17 | 1.4 (0.9-2.3) | 0.18 |
| Sarcoma | 0.5 (0.2-1.1) | 0.07 | 0.5 (0.2-1.1) | 0.07 | 0.5 (0.2-1.1) | 0.07 |
| Testicular/Ovarian | 1.0 (0.7-1.6) | 0.85 | 1.0 (0.7-1.6) | 0.86 | 1.0 (0.7-1.6) | 0.84 |
| Breast | Ref | - | Ref | - | Ref | - |
| Colorectal | 1.6 (0.9-2.8) | 0.08 | 1.6 (1.0-2.9) | 0.07 | 1.7 (1.0-2.9) | 0.07 |
| Thyroid | 1.0 (0.6-1.6) | 0.84 | 0.9 (0.6-1.6) | 0.84 | 1.0 (0.6-1.6) | 0.86 |
| Other | 1.1 (0.7-1.7) | 0.65 | 1.1 (0.7-1.7) | 0.65 | 1.1 (0.7-1.7) | 0.61 |
| Region |  |  |  |  |  |  |
| Central | 0.9 (0.6-1.2) | 0.45 | 0.9 (0.6-1.2) | 0.46 | 0.9 (0.6-1.2) | 0.48 |
| East | *1.4 (1.0-2.0)* | *0.05* | *1.4 (1.0-2.0)* | *0.05* | *1.4 (1.0-2.0)* | *0.04* |
| North | *1.6 (1.1-2.5)* | *0.02* | *1.6 (1.1-2.4)* | *0.03* | *1.6 (1.1-2.5)* | *0.02* |
| Toronto | Ref | - | Ref | - | Ref | - |
| West | 1.0 (0.7-1.4) | 0.89 | 1.0 (0.7-1.4) | 0.89 | 1.0 (0.7-1.5) | 0.85 |
| Previous mental health- related outpatient visit |  |  |  |  |  |  |
| Yes | **2.9 (2.4-3.5)** | **<0.001** | **2.9 (2.4-3.5)** | **<0.001** | **2.9 (2.4-3.6)** | **<0.001** |
| No | Ref | - | Ref | - | Ref | - |
| Previous SMHE |  |  |  |  |  |  |
| Yes | **2.8 (2.2-3.8)** | **<0.001** | **2.8 (2.2-3.8)** | **<0.001** | **2.9 (2.2-3.8)** | **<0.001** |
| No | Ref | - | Ref | - | Ref | - |
| ESAS Score |  |  |  |  |  |  |
| Not measured | 1.3 (0.9-1.8) | 0.17 | 1.3 (0.9-1.8) | 0.17 | 1.2 (0.8-1.7) | 0.32 |
| Mild | Ref | - | Ref | - | Ref | - |
| Moderate | **5.1 (3.1-8.5)** | **<0.001** | **3.4 (2.0-5.8)** | **<0.001** | **3.0 (1.9-4.9)** | **<0.001** |
| Severe | **3.5 (1.7-7.3)** | **<0.001** | **3.6 (1.9-6.7)** | **<0.001** | 2.2 (1.0-4.9) | 0.06 |

Italicized hazard ratios indicate p<0.05 while bolded hazard ratios indicate p<0.001.

HR – hazard ratio; SMHE - severe mental health event

Supplemental Table 3. Distributions of demographic and disease characteristics of subcohort with at least five years of follow-up (N=3,518)

|  | **N (%)** |
| --- | --- |
| Age (years) (median, IQR) | 25 (21-27) |
| Sex |  |
| Male | 1825 (51.9) |
| Female | 1693 (48.1) |
| Time period |  |
| Early (2010-2014) | 2,729 (77.6) |
| Late (2015-2018) | 789 (22.4) |
| Neighborhood income quintile |  |
| Rural | 328 (9.3) |
| Urban Q1 (lowest) | 576 (16.4) |
| Urban Q2 | 643 (18.3) |
| Urban Q3 | 640 (18.2) |
| Urban Q4 | 659 (18.7) |
| Urban Q5 (highest) | 659 (18.7) |
| Cancer type |  |
| Hematologic | 1,159 (32.9) |
| Melanoma | 238 (6.8) |
| CNS | 182 (5.2) |
| Sarcoma | 124 (3.5) |
| Testicular/Ovarian | 824 (23.4) |
| Breast | 211 (6.0) |
| Colorectal | 118 (3.4) |
| Thyroid | 274 (7.8) |
| Other | 388 (11.0) |
| Region |  |
| Central | 1,162 (33.0) |
| East | 792 (22.5) |
| North | 218 (6.2) |
| Toronto | 389 (11.1) |
| West | 957 (27.2) |

CNS – central nervous system; IQR – interquartile range; N – number

Supplemental Table 4. Multivariable predictors of late severe mental health events

|  | Depression | | Anxiety | | Wellbeing | |
| --- | --- | --- | --- | --- | --- | --- |
|  | Adjusted HR | P-value | Adjusted HR | P-value | Adjusted HR | P-value |
| Age (per 10 years) | 0.8 (0.5-1.2) | 0.24 | 0.8 (0.5-1.2) | 0.24 | 0.8 (0.5-1.2) | 0.23 |
| Sex |  |  |  |  |  |  |
| Male | Ref | - | Ref | - | Ref | - |
| Female | 1.2 (0.8-1.7) | 0.42 | 1.2 (0.8-1.7) | 0.41 | 1.2 (0.8-1.7) | 0.38 |
| Time period |  |  |  |  |  |  |
| Early (2010-2014) | 1.5 (0.7-3.1) | 0.25 | 1.5 (0.7-3.0) | 0.27 | 1.5 (0.7-3.0) | 0.27 |
| Late (2015-2018) | Ref | - | Ref | - | Ref | - |
| Neighborhood income quintile |  |  |  |  |  |  |
| Rural | 1.6 (0.8-3.1) | 0.15 | 1.5 (0.8-2.9) | 0.19 | 1.6 (0.8-3.0) | 0.18 |
| Urban Q1 (lowest) | *1.8 (1.0-3.2)* | *0.04* | *1.8 (1.0-3.1)* | *0.05* | 1.7 (1.0-3.1) | 0.06 |
| Urban Q2 | 1.5 (0.8-2.7) | 0.16 | 1.4 (0.8-2.5) | 0.23 | 1.4 (0.8-2.6) | 0.20 |
| Urban Q3 | 1.2 (0.7-2.1) | 0.60 | 1.1 (0.6-2.0) | 0.74 | 1.2 (0.6-2.1) | 0.61 |
| Urban Q4 | 1.2 (0.6-2.1) | 0.64 | 1.1 (0.6-2.1) | 0.66 | 1.1 (0.6-2.0) | 0.69 |
| Urban Q5 (highest) | Ref | - | Ref | - | Ref | - |
| Cancer type |  |  |  |  |  |  |
| Hematologic | 1.0 (0.5-2.0) | 0.97 | 1.0 (0.5-2.1) | 0.99 | 1.0 (0.5-2.0) | 0.92 |
| Melanoma | 1.1 (0.5-2.7) | 0.83 | 1.1 (0.4-2.6) | 0.88 | 1.1 (0.5-2.7) | 0.81 |
| CNS | 1.1 (0.4-2.8) | 0.92 | 1.0 (0.4-2.7) | 0.97 | 1.0 (0.4-2.6) | 1.0 |
| Sarcoma | 1.1 (0.4-3.3) | 0.87 | 1.2 (0.4-3.5) | 0.78 | 1.1 (0.4-3.2) | 0.90 |
| Testicular/Ovarian | 1.0 (0.4-2.2) | 0.98 | 1.0 (0.5-2.2) | 0.99 | 1.0 (0.5-2.2) | 1.0 |
| Breast | Ref | - | Ref | - | Ref | - |
| Colorectal | 1.2 (0.4-3.5) | 0.78 | 1.2 (0.4-3.5) | 0.76 | 1.2 (0.4-3.6) | 0.73 |
| Thyroid | 1.0 (0.4-2.3) | 0.97 | 1.0 (0.4-2.3) | 0.98 | 1.0 (0.5-2.4) | 0.94 |
| Other | 1.0 (0.5-2.3) | 0.94 | 1.0 (0.5-2.2) | 0.96 | 1.1 (0.5-2.3) | 0.87 |
| Region |  |  |  |  |  |  |
| Central | 1.1 (0.6-2.0) | 0.84 | 1.0 (0.5-2.0) | 0.92 | 1.0 (0.6-2.0) | 0.89 |
| East | 1.9 (1.0-3.5) | 0.05 | 1.8 (1.0-3.4) | 0.06 | *1.9 (1.0-3.5)* | *0.05* |
| North | *2.2 (1.0-4.6)* | *0.04* | 2.0 (0.9-4.2) | 0.07 | *2.2 (1.0-4.5)* | *0.04* |
| Toronto | Ref | - | Ref | - | Ref | - |
| West | 1.1 (0.6-2.1) | 0.82 | 1.0 (0.5-2.0) | 0.92 | 1.1 (0.6-2.1) | 0.84 |
| Previous mental health- related outpatient visit |  |  |  |  |  |  |
| Yes | **1.9 (1.3-2.8)** | **<0.001** | **2.0 (1.4-2.9)** | **<0.001** | **2.1 (1.5-3.0)** | **<0.001** |
| No | Ref | - | Ref | - | Ref | - |
| Previous SMHE |  |  |  |  |  |  |
| Yes | **4.2 (2.6-6.7)** | **<0.001** | **4.2 (2.6-6.7)** | **<0.001** | **4.3 (2.7-6.8)** | **<0.001** |
| No | Ref | - | Ref | - | Ref | - |
| ESAS Score |  |  |  |  |  |  |
| Not measured | **1.8 (1.2-2.8)** | **0.004** | **1.9 (1.2-2.9)** | **0.006** | **2.4 (1.5-4.1)** | <**0.001** |
| Mild | Ref | - | Ref | - | Ref | - |
| Moderate | **2.4 (1.5-3.9)** | **<0.001** | 1.6 (1.0-2.7) | 0.07 | **3.2 (1.9-5.2)** | **<0.001** |
| Severe | **3.0 (1.8-4.9)** | **<0.001** | **2.8 (1.7-4.6)** | **<0.001** | **2.6 (1.5-4.5)** | **0.001** |

Italicized hazard ratios indicate p<0.05 while bolded hazard ratios indicate p<0.001.

HR – hazard ratio; SMHE - severe mental health event

Supplemental Table 5. Number and percentage of patients with late SMHE* within cohort subgroups

A. Anxiety

| Symptom burden | Pre-cancer diagnosis outpatient mental health use | Pre-cancer diagnosis SMHE | Number (percentage) of full cohort | Number (percentage) of all late SMHE |
| --- | --- | --- | --- | --- |
| Not severe | - | - | 3058 (86.9) | 89 (76.2) |
| Severe | - | - | 460 (13.1) | 31 (23.8) |
| Not severe | No | - | 2486 (70.7) | 55 (45.8) |
| Not severe | Yes | - | 572 (16.3) | 34 (28.3) |
| Severe | No | - | 303 (8.6) | 10 (8.3) |
| Severe | Yes | - | 157 (4.5) | 21 (17.5) |
| Not severe | - | No | 2965 (84.3) | 71 (59.2) |
| Not severe | - | Yes | 93 (2.6) | 18 (15.0) |
| Severe | - | No | 432 (12.3) | 25 (20.8) |
| Severe | - | Yes | 28 (0.8) | 6 (5.0) |

B. Depression

| Symptom burden | Pre-cancer diagnosis outpatient mental health use | Pre-cancer diagnosis SMHE | Number (percentage) of full cohort | Number (percentage) of all late SMHE |
| --- | --- | --- | --- | --- |
| Not severe | - | - | 3250 (92.4) | 99 (82.5) |
| Severe | - | - | 268 (6.7) | 21 (17.5) |
| Not severe | No | - | 2631 (74.8) | 58 (48.3) |
| Not severe | Yes | - | 619 (17.6) | 41 (34.2) |
| Severe | No | - | 158 (4.5) | 7 (5.8) |
| Severe | Yes | - | 110 (3.1) | 14 (11.7) |
| Not severe | - | No | 3151 (89.6) | 81 (67.5) |
| Not severe | - | Yes | 99 (2.8) | 18 (15.0) |
| Severe | - | No | 246 (7.0) | 15 (12.5) |
| Severe | - | Yes | 22 (0.6) | 6 (5.0) |

N – Number; SMHE – Severe mental health event

*In this table, defined as SMHE occurring between 5- and 8- years following cancer diagnosis.

**Appendix 1**


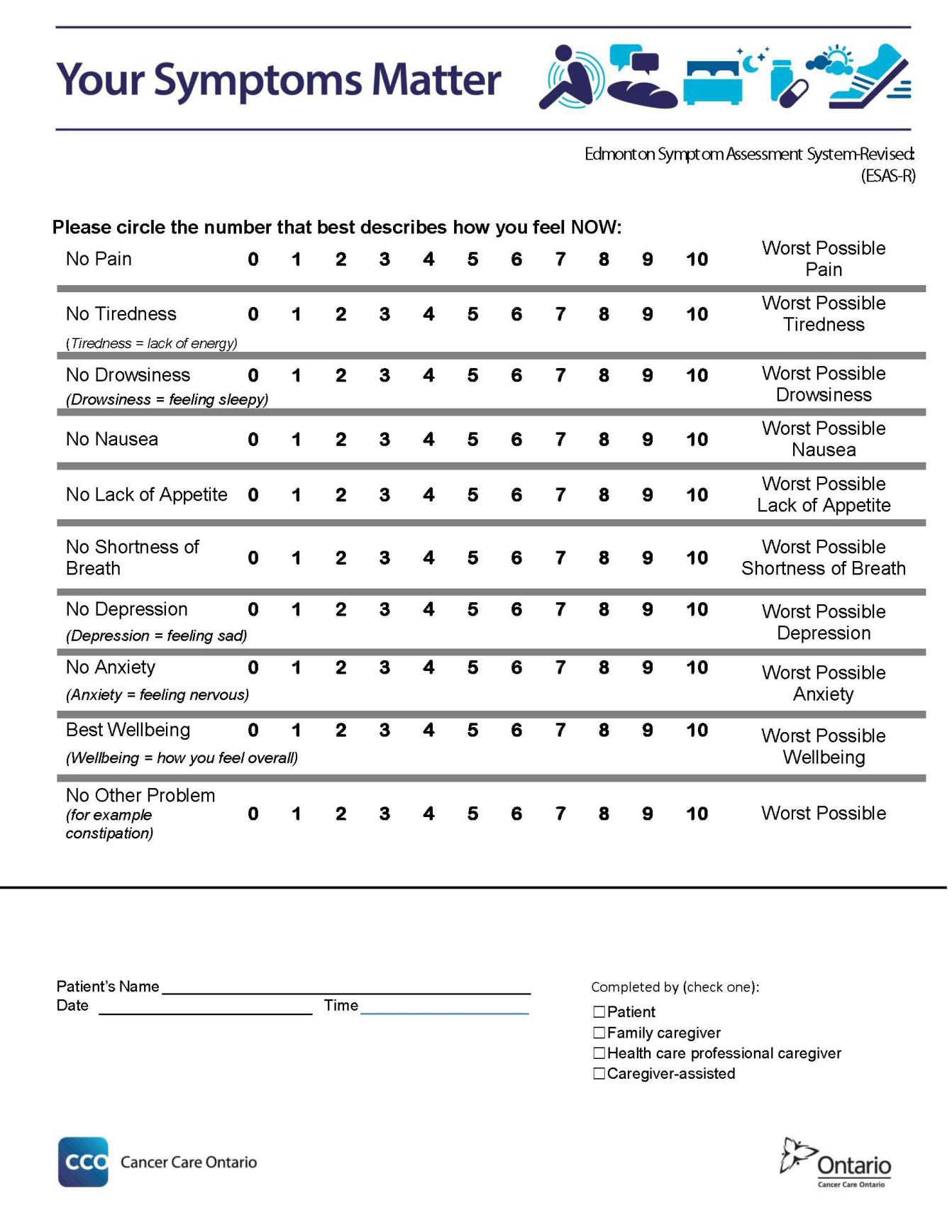


**Appendix 2 – Algorithms used to determine mental health encounters**

**FAMILY PHYSICIAN VISIT**

Outpatient visit with a mental health diagnostic code labeled with a specialty identifier of general physician were extracted from OHIP (OHIP spec = "00"). Any lab fee codes were excluded. A modified Steele’s algorithm^12^ was utilized and data was identified according to the following OHIP codes.

**Mental Health Diagnostic Codes:**

***Psychotic Disorders***

295 Schizophrenia

296 Manic-depressive psychoses, involutional melancholia

297 Other paranoid states

298 Other psychoses

***Non-Psychotic Disorders***

300 Anxiety neurosis, hysteria, neurasthenia, obsessive-compulsive neurosis, reactive depression

301 Personality disorders

302 Sexual deviations

306 Psychosomatic illness

309 Adjustment reaction

311 Depressive disorder

***Substance Use Disorders***

303 Alcoholism

304 Drug dependence

**OUTPATIENT PSYCHIATRIST VISIT**

A visit to a psychiatrist was defined as any outpatient visit codes submitted by a psychiatrist (OHIP spec = "19"). Lab fee codes were excluded.

**EMERGENCY DEPARTMENT VISIT**

A visit to the emergency department was captured through both OHIP (before 2002) and NACRS (2002 and after) depending on the years of mental health-related service utilization.

**OHIP Diagnostic Codes Before 2002:**

Billings submitted with var location = "E", spec = "12", and/or combination of the diagnostic codes:

291 Alcoholic psychosis, delirium tremens, Korsakov's psychosis

292 Drug psychosis

295 Schizophrenia

296 Manic-depressive psychoses, involutional melancholia

297 Other paranoid states

298 Other psychoses

300 Anxiety neurosis, hysteria, neurasthenia, obsessive-compulsive neurosis, reactive depression

301 Personality disorders

302 Sexual deviations

303 Alcoholism

304 Drug dependence

305 Tobacco abuse

306 Psychosomatic illness

307 Habit spasms, tics, stuttering, tension headaches, anorexia nervosa, sleep disorders, enuresis

309 Adjustment reaction

311 Depressive disorder

319 Mental retardation

**NACRS 2002 Onwards:**

From NACRS (2001-2013). DX10CODE1 with any of the following Canadian Coding Standards of the International Classification of Diseases, Tenth Revision (ICD-10-CA), codes or DXCODE1 (2001-2013):

***Overall***

ICD-10-CA: F10to F99 OR: X60-84, Y10-Y19, Y28 in Dx10Code2 to Dx10Code10 and no specified Mental Health code in Dx10Code1 (F04-F99)

ICD-9-CA: 290-319

***Substance-Related Disorders (SAs)***

ICD-10-CA: F55, F10 to 19F in Dx10Code1

ICD-9-CA: 291.0-291.9, 292.0-292.9, 303.0-303.9, mental health code (F04- F99) in Dx10Code1

***Schizophrenia, Delusional and Nonorganic Psychotic Disorders (SCZs)***

ICD-9-CA: 295.00-295.95, 297.0-297.2, 297.8, 297.9, 298.3, 298.4, 298.8, 298.9, 297.3, 295.50-295.75, 298.1, 293.89

ICD-10-CA: F20 (excluding F20.4), F22, F23, F24, F25, F28, F29, F53.1 in Dx10Code1

***Mood/affective disorders (MAs)***

ICD-10-CA: F30, F31, F32, F33, F34, F38, F39, F53.0 in Dx10Code1

ICD-9-CA: 296.01-296.06, 296.10-296.16, 296.81, 296.40-296.46, 296.50-296.56, 296.60-296.66, 296.7, 296.89, 296.80, 296.21-296.26, 298.0, 296.82, 296.20, 311, 296.30-296.36, 296.99, 300.4,

301.10, 301.12, 301.13, 296.90

***Anxiety and adjustment disorders (ANXs)***

ICD-10-CA: F40, F41, F42, F43, F48.8, F48.9 in Dx10Code1

ICD-9-CA: 300.20-300.23, 300.29, 300.00-300.02, 300.09, 300.3, 308.0-308.4, 308.9, 309.0, 309.1, 309.24, 309.28, 309.29, 309.3, 309.4, 309.82, 309.83, 309.89, 300.5, 300.9, 300.89, V402

***Neurodevelopmental and Other Selected Disorders (Other)***

ICD-10-CA: F60–F62, F68, F69, F21, F80–F84, F88-F92, F93 (excluding F93.1, F93.2), F94, F95, F98 in Dx10Code1

ICD-9-CA: 301.0, 301.20, 301.21, 301.7, 301.3, 301.81-301.84, 301.50, 301.59, 301.4, 301.6, 301.11, 301.89, 301.9, 300.16, 300.19, 301.51, V403.9, V409, 301.22

***Self-Harm ED Visits without an MHA Diagnosis (NACRS only)***

(X60-X84, Y10-Y19, Y28) in Dx10Code2 to Dx10Code10 and no specified mental health code in Dx10Code1 (F04 to F99)

***Intentional Self-Harm***

X60-X84, Y10-Y19, Y28 in Dx10Code2-x10Code10304.0-304.9, 305.0-305.9 and no other specified

**HOSPITALIZATIONS**

Mental health related hospitalizations were derived from DAD and OMHRS. From DAD var DX10CODE1 with any of the following ICD-10-CA codes were utilized for diagnoses from 2002 on. From 1992 to 2001, DXCODE1 with any of the following ICD-9-CA codes were pulled.

From OMHRS, DSM-IV were extracted.

***Overall***

ICD-9-CA: 290-319

ICD-10-CA: DX10CODE1 = F10 to F99,

or DX10CODE2 to DX10CODE10 = X60-X84, Y10-Y19, Y28 AND DX10CODE1 ne F04 to F99

DSM-IV: Any (including missing diagnoses; excluding 290.x or 294.x)

***Substance-related disorders (SA)***

ICD-9-CA: 291.0-291.9, 292.0-292.9, 303.0-303.9, 304.0-304.9, 305.0-305.9

ICD-10-CA: F55, F10 to F19

DSM-IV: 291.x (all 291 codes, excluding 291.82), 292.x (all 292 codes, excluding 292.85), 303.x (all 303 codes), 304.x (all 304 codes), 305.x (all 305 codes)

PROVDX1: 4

***Schizophrenia, delusional and non-organic psychotic disorders (SCZ)***

ICD-9-CA: 295.00-295.95, 297.0-297.2, 297.8, 297.9, 298.3, 298.4, 298.8, 298.9, 297.3, 295.50-295.75, 298.1, 293.89

ICD-10-CA: F20 (excluding F20.4), F22, F23, F24, F25, F28, F29, F53.1

DSM-IV: 295.x (all 295 codes), 297.x (all 297 codes), 298.x (all 298 codes)

PROVDX1: 5

***Mood/affective disorders (MA)***

ICD-9-CA: 296.01-296.06, 296.10-296.16, 296.81, 296.40-296.46, 296.50-296.56, 296.60-296.66, 296.7, 296.89, 296.80, 296.21-296.26, 298.0, 296.82, 296.20, 311, 296.30-296.36, 296.99, 300.4, 301.10, 301.12, 301.13, 296.90, 293.89

ICD-10-CA: F30, F31, F32, F33, F34, F38, F39, F53.0

DSM-IV: 296.x (all 296 codes), 300.4x, 301.1

PROVDX1: 6

***Anxiety and adjustment disorders (ANX)***

ICD-9-CA: 300.20-300.23, 300.29, 300.00-300.02, 300.09, 300.3, 308.0-308.4, 308.9, 309.0, 309.1, 309.24, 309.28, 309.29, 309.3, 309.4, 309.82, 309.83, 309.89, 300.5, 300.9, 300.89, V402

ICD-10-CA: F40, F41, F42, F43, F48.8, F48.9, F93.1, F93.2

DSM-IV: 300, 300.0x, 300.2x, 300.3x, 308.3x, 309.0x, 309.24, 309.28, 309.3x, 309.4x, 309.8x, 309.9x

PROVDX1: 7, 15

***Selected disorders of adult personality and behavior (Other)***

ICD-9-CA: 301.0, 301.20, 301.21, 301.7, 301.3, 301.81-301.84, 301.50, 301.59, 301.4, *301.6, 301.11, 301.89, 301.9, 300.16, 300.19, 301.51, V403.9, V409, 301.22*

ICD-10-CA: F60, F61, F62, F68, F69, F21

DSM-IV:300.16, 300.19, 301.x (all 301 codes excluding 301.1x)

PROVDX1: 1,16

***Short stay at psychiatric hospital (< 72 days)***

ICD-10-CA: n/a

DSM-IV: Admission count required

***Intentional Self-Harm (SH)***

ICD-10-CA: X60-X84, V01, W00, W20-W65, W85, X00, X20-X29, X40, X50

DSM-IV: n/a

Where DXTYPE= 9, ICD-9-CA: E950-E959, E980-E989, E810-E848, E850-E928

***Deliberate Self-Harm hospitalizations without a MHA diagnosis (DSH); DAD only***

ICD-10-CA: Dx10Code2 to Dx10Code10 = X60-X84, Y10-Y19, Y28 and no specified Mental Health code in Dx10Code1 (F04 to F99)

**Appendix 3 – Details of Cohort Creation**

Of 9,399 identified patients, 649 (6.9%) were diagnosed and treated at pediatric institutions. Of the 8,750 AYA diagnosed and treated at adult institutions, 2,949 (33.7%) had no cancer-related visit within the first year of diagnosis, as indicated by the Activity Level Reporting (ALR) database, and were thus excluded. The ALR database includes data on patient level cancer-related activity (e.g. radiation and systemic therapies, outpatient oncology clinic visits). Confirming previous assumptions that such patients represent cancer diagnoses that do not require services at a cancer center, 1,416 (48.0%) had thyroid cancer, only 47 died within a year of diagnosis, and only 82 were ESAS screened within a year. Of the remaining 5,801 patients with ALR visits within the first year, 366 (6.3%) only had ALR visits at non-RCCs and thus also excluded. These excluded patients were less likely to live in rural areas [13/366 (3.6%) vs. 535/5435 (9.8%); p<0.001] and more likely to be diagnosed in the later time period [242/366 (66.1%) vs. 2,175/5,435 (40.0%); p<0.001].
